# *Ex vivo* assessment of bicuspidization repair in treating severe functional tricuspid regurgitation: a stereo-scopic PIV study

**DOI:** 10.1038/s41598-019-47873-3

**Published:** 2019-08-08

**Authors:** Yen Ngoc Nguyen, Foad Kabinejadian, Munirah Ismail, William Kok-Fai Kong, Edgar Lik Wui Tay, Hwa Liang Leo

**Affiliations:** 1 Department of Biomedical Engineering, National University of Singapore, Singapore, Singapore; 2 Department of Biomedical Engineering, Tulane University, New Orleans, LA 70112 USA; 3 School of Mechanical and Aerospace Engineering, Nanyang Technological University, Singapore, Singapore; 4 Department of Cardiology, National University Heart Centre, Singapore, Singapore; 5 NUS Graduate School for Integrative Sciences and Engineering, National University of Singapore, Singapore, Singapore

**Keywords:** Cardiac device therapy, Biomedical engineering

## Abstract

There has been a resurgence of interest in the treatment of severe functional tricuspid regurgitation (FTR) due to the awareness of its poor outcomes and potential percutaneous therapies. Kay bicuspidization has been adapted in percutaneous therapies but its clinical outcome remains uncertain. The present study evaluates the efficacy of Kay repair in a novel *ex vivo* pulsatile system. Porcine tricuspid valve (TV) (n = 3) was extracted and incorporated into a patient-specific silicon right ventricle (RV) emulating severe FTR, on which Kay repair was subsequently performed. TV area metrics and RV hemodynamic assessment by means of stereo-scopic particle image velocimetry were quantified in both FTR and post-repair conditions. Bicuspidization led to significant increase in cardiac output although the overall increment due to this approach alone was generally small, possibly due to existence of residual TR and the large reduction in TV opening area. Kinetic energy and viscous loss levels were increased post-repair, especially during diastolic filling. Main vortex structures generally maintained post-procedural. However, there was enhanced swirling motion in larger RV domain. Although this might reduce mural-thrombus risk, the relatively more complex vortex phenomenon likely resulted in elevated viscous loss observed and may potentially impact long-term adaptation. The RV hemodynamic alteration after tricuspid repair could be used to predict the success of these future transcatheter solutions.

## Introduction

The most common mechanism of clinically relevant tricuspid regurgitation (TR) is functional TR (FTR) in which right ventricular (RV) enlargement and/or tricuspid annular (TA) dilatation result in TV malfunction despite the intact TV apparatus^1^. Severe TR can contribute to right heart failure and increased mortality rate^2^. In clinical practice, more aggressive FTR management has been recommended after realisation of its progressive nature and poor prognosis^3^. Especially, the evolving transcatheter technology has allowed the implementation of different surgical repair and replacement therapies by percutaneous approach. However, it remains a great topic of debate regarding the timing, diagnosis and the choice of FTR treatment^4^. One of the common FTR surgical treatment options is Kay bicuspidization repair, in which the 2 points at annular region along the posterior leaflet is apposed together by sutures, essentially plicating the posterior leaflet^5^. This leads to a double-leaflet configuration formed by the anterior and septal leaflets, thus the name “bicuspidization”. This technique is of special interest to the interventional community as it is a method of repair adapted into future percutaneous repair technology.

However, alteration of the natural TV apparatus by bicuspidization repair might alter RV fluid dynamics and energy behaviour. Many studies have demonstrated better hemodynamic and energy behaviour associated with physiological vortex and flow patterns compared to other pathologies or procedures^6^. There are benefits in studying such flow patterns; e.g., flow alteration after different therapies could provide an early surrogate for cardiovascular outcomes^7^ and potentially be a therapeutic target or assessment tool. While numerous studies have looked into the LV flows, those done on the RV are extremely limited. Compared to the left ventricle, the RV has a more irregular geometrical shape, is an asymmetric structure with more complex TV morphology. This makes *in vivo* imaging access more challenging and computational modelling susceptible to certain sources of errors. To date, a few *in vivo*^8–11^ and *in silico*^12,13^ studies have looked into the details of physiological RV flow, and there is yet no attempt to quantify RV hemodynamics after different surgical procedures such as those done on atrio-ventricular valve.

In this study, a novel *ex vivo* mock circulatory system was set up to investigate the three-dimensional (3D) RV flow by stereo-scopic particle image velocimetry (PIV). Hemodynamic characteristics and energy parameters inside a patient-specific silicon RV model, which incorporated an extracted porcine valve, were analyzed. Two scenarios were investigated: (i) severe FTR, and (ii) after TV bicuspidization repair. In addition, quantification of different TV area metrics was performed. To the best of our knowledge, this is the first attempt to characterize the RV hemodynamics after any TV repair therapy.

## Materials and Methods

### Patient-specific RV model and pulmonary valve

**Figure 1:**
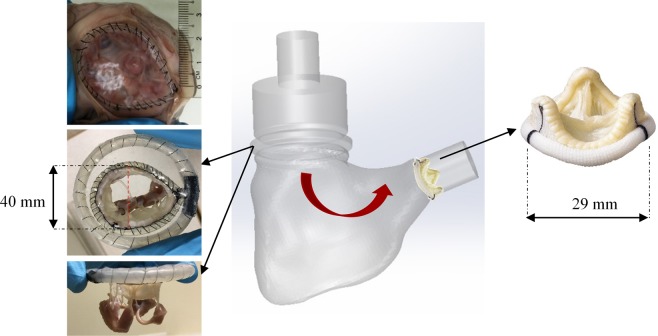
Patient-specific RV model with a circular circumferential slot at TV position to accommodate TV annular ring, red arrow denotes the main direction of forward flow. St. Jude Epic bioprosthetic valve was incorporated at the pulmonary outlet.

The construction of a transparent RV silicon model based on severe FTR patient-specific geometry has been described before^14^. The RV was modified in order to include a circular circumferential track to be fitted with TV annular ring (Fig. 1). TV annular ring was made up of a thin and flexible silicon membrane, where a porcine extracted TV could be sutured upon. A St. Jude Medical Epic tri-leaflet tissue valve of 29 mm diameter was sutured at the pulmonary outlet of the ventricle to function as a pulmonary valve (Fig. 1).

### Regurgitant and repaired tricuspid valves

Fresh porcine hearts were collected from a local slaughterhouse. The right atrial appendage was cut off to provide visual exposure of the tricuspid orifice. RV cavity was pressurized by water in static condition to assess the TV condition. Upon examination of the hearts, those possessing proper TV closure and TV dimensions within human physiological ranges were selected^15,16^.

A thin flexible Tygon tubing was sutured along the tricuspid orifice, preserving the natural shape and dimension of the TV (Fig. 1). Subsequently, the TV apparatus was excised with its chordae tendineae and papillary muscle (PM) groups intact. PM groups were slightly treated with glutaraldehyde (0.5%) for 2 minutes to increase the resistance to stitching. The TV apparatus was then sutured onto the mentioned TV annular membrane. The anterior-septal diameter was increased to 40 mm (Fig. 1) to model significant TV annular dilatation^16^. The whole annular ring housing the TV apparatus was slotted into the circular track inside the silicon RV. The PMs were sutured onto the RV wall according to their anatomical positions, approximately below the three commissural points. To account for the anatomical variations, three valve samples were re-constructed, for which hemodynamic conditions before and after bicuspidization repair were examined. The valves (i) in FTR state and (ii) after bicuspidization repair are referred to as group (a) and group (b), respectively.

**Figure 2:**
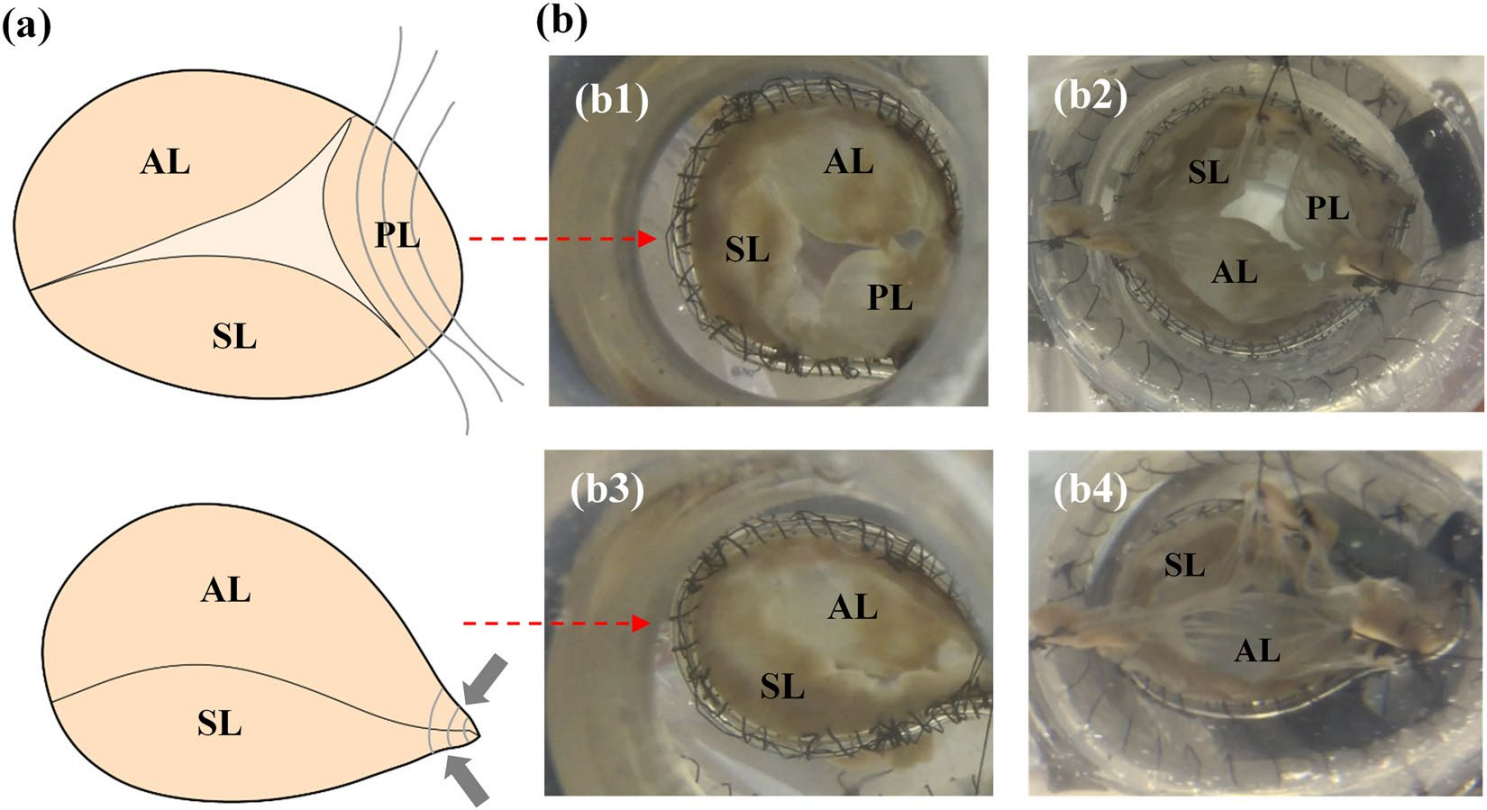
(**a**) Kay Bicuspidization strategy: posterior leaflet is excluded by plicating the annulus sections along posterior leaflet using sutures. (**b**) Valve 1a (FTR) leaflet configuration at peak systole from atrial view (b1) and ventricular view (b2); Valve 1b (bicuspidization repair) leaflet configuration at peak systole from atrial view (b3) and ventricular view (b4). AL, anterior leaflet; SL, septal leaflet; PL, posterior leaflet.

Due to the severe dilatation of both RV and TV annulus, the valves in FTR state exhibited severe regurgitation. As seen in Fig. 2, both atrial and ventricular views of Valve 1a orifice at peak systole show incomplete coaptation with a significant central gap. After conducting experiments on the three valves in their pathological conditions, Kay bicuspidization repair was performed on each valve based on established technique^5^. In summary, the posterior leaflet was obliterated by plicating the annulus along this leaflet. 5–0 surgical sutures were used to tighten and secure both sides of the annulus together as depicted in Fig. 2. This modification essentially reduced the TV orifice area and converted the three-leaflet morphology to bi-leaflet configuration, somewhat similar to native MV.

### Flow loop setup

**Figure 3:**
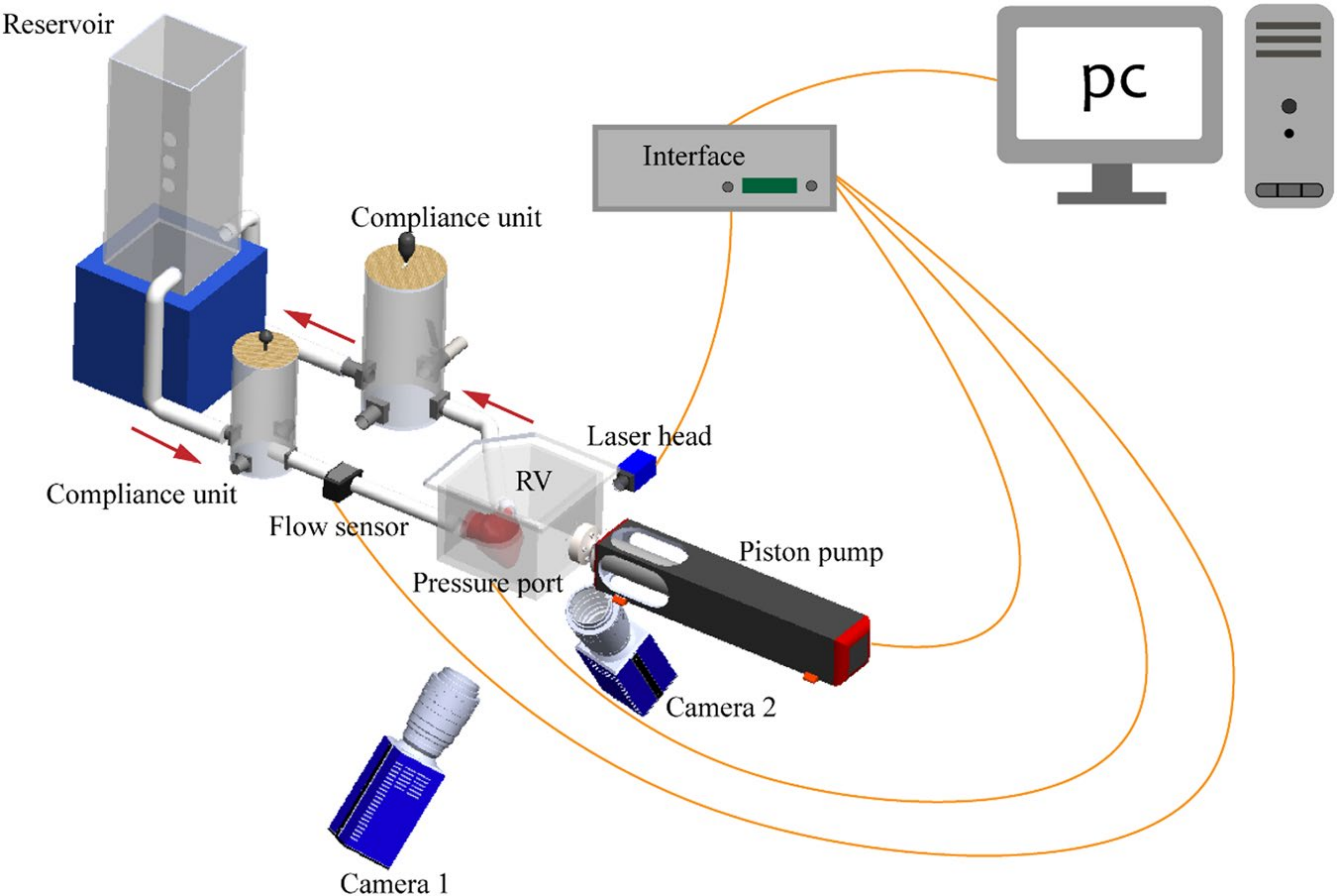
Schematic diagram of the mock circulatory system and PIV setup.

A right heart simulator was developed previously for RV flow assessment^14^. The system configuration was designed in order to allow optical access and laser illumination in compatibility with stereo-scopic PIV (Fig. 3). Blood analogue was made by mixing glycerine and water with the volume ratio of approximately 40:60^17,18^, resulting in average density of 1081 kg/m^3^ and average kinematic viscosity of 4.02 cP.

**Figure 4:**
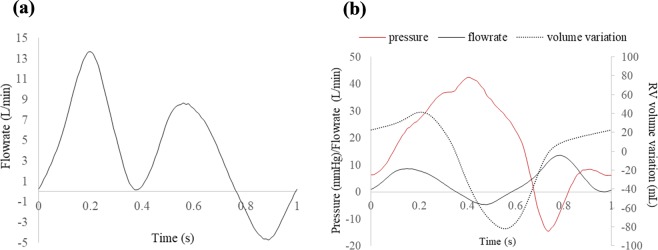
(**a**) Trans-tricuspid flow curve of regurgitant Valve 1a; (**b**) RV pressure, trans-tricuspid flow and RV volume variation curves in a cardiac cycle for native regurgitant Valve 1a.

Trans-tricuspid flow waveform and cardiac output (CO) were measured using clamp-on flow sensor (ME16PXL, Transonic Systems Inc., Ithaca, NY). RV pressure was recorded using a pressure catheter (Mikro-Tip SPR-340S, Millar Instruments Inc., Houston, TX, USA). In this study, the simulator was calibrated to emulate severe FTR condition, characterized by elevated RV pressure, more retrograde flow, and mean CO of 3.9 L/min. Calibrated hemodynamic waveforms for Valve 1 are depicted in Fig. 4, and average hemodynamic parameters for all the valves before and after repair are summarized in Table 1. Heartbeat and stroke volume were fixed at 60 beats/minute and 83.3 mL/heartbeat, respectively.

**Table 1:** Flow and pressure parameters of the two valve groups.

	Cardiac output (L/min)	Peak flow rate (L/min)	Peak RV pressure (mmHg)
FTR (n = 3)	3.90 ± 0.00	13.68 ± 0.17	42.88 ± 1.21
Bicuspidization (n = 3)	4.13 ± 0.06	13.41 ± 0.16	44.87 ± 0.78

Results are represented as mean ± SD.

FTR = functional tricuspid regurgitation; RV = right ventricle.

### Stereo-scopic PIV

The working fluid was seeded with polyamide particles (PSP-20, Dantec Dynamis, Skovlunde, Denmark) with density of 1.03 g/cm^3^, refractive index of 1.5 and mean diameter of 20 µm. Particles on the plane of interest were illuminated with a laser sheet emitted from a 15 Hz Q-switched, pulsed Nd:YAG laser source (Nano S35 PIV, Litron Lasers, Rugby, UK). The thickness of laser sheet was kept at ~3 mm. Two synchronized CCD cameras (Imager pro X, LaVision, Germany) were used to acquire 3D velocity components of the flow (Fig. 3). A Scheimpflug adapter (Model 1108196, LaVision, Germany) was fitted on each camera to aid proper focusing on the region of interest. The in-plane spatial resolution of the dewarped image was at 1677 × 1230 pixel^2^, which corresponded to the physical domain of approximately 124 × 92 mm^2^ for each measurement plane.

To construct a volumetric flow velocity field, PIV was measured at 22 parallel planes along the long-axis of RV, spaced 3 mm apart. The PIV measurement time-points were phase-locked by synchronization with the trans-tricuspid flow waveform, such that recording was triggered at a particular time instance during a cardiac cycle. 150 measurements at each time instant were found to be sufficient to obtain statistical convergence of a stable velocity vector map^14,19^. Measurements were then repeated for 14 different time points in a cycle.

To derive the 3D velocity field from particle images, stereo cross-correlation was performed with Davis 8.3.1 (LaVision GmbH, Goettingen, Germany). Multi-pass iteration scheme with decreasing interrogation window size was used, resulting in the final interrogation window size of 64 × 64 pixels with 50% overlap. This method yielded one velocity vector for every grid area of 2.4 × 2.4 mm^2^. Post-processing of the derived velocity vector field was performed with built-in median filters and smoothing schemes in Davis 8.3.1 software. The flow velocity field was then interpolated by Kriging algorithm to yield a full three-dimensional visualization and assessment.

### Valve area assessment

**Figure 5:**
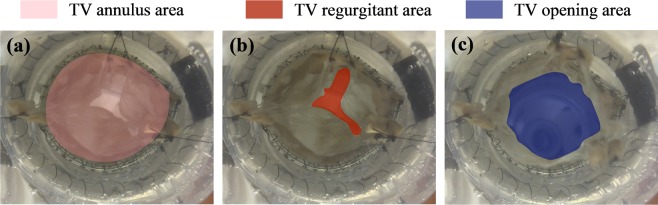
Tricuspid valve area quantification shown for Valve 1a during coaptation (**a**,**b**), and during peak opening instant (**c**) using ventricular view images. Three distinct colour codes are used to denote annulus area (**a**), regurgitant area (**b**), and opening area (**c**), respectively.

The performance of the valves before and after surgical repair was recorded in both ventricular view and atrial view over a cardiac cycle. Image processing by ImageJ was then performed at the peak opening instant and peak systolic instant to quantify the TV annulus area, TV opening area (the narrowest part of the TV, measured at the maximal valve opening instant), and TV regurgitant area (the central gap of the TV, measured at the peak systole) (Fig. 5).

### Energy analysis

#### Mean kinetic energy (MKE)

Mean kinetic energy (MKE) was obtained from the mean velocity component at every spatial point in the RV at each time-step as follows:

1 $${MKE}=\frac{1}{2}\rho \cdot {|\bar{{\boldsymbol{v}}}|}^{2}(N/{m}^{2})$$

Where *ρ* (kg/m^3^) is the density of the working fluid, $\bar{\nu }$ is the ensemble average velocity vector of the fluid, taken as the average of 150 instantaneous velocity measurements over 150 cycles. Total average MKE over a cardiac cycle was taken as the sum of the average MKE at all time-instants recorded.

#### Viscous energy loss (VEL)

Viscous dissipation energy loss (VEL) is the irreversible mechanical kinetic energy loss due to frictional forces, mainly in the form of thermal energy^20^. Viscous dissipation function Φ_v_ which represents the rate of viscous energy dissipation per unit volume was computed as:

$${{\rm{\Phi }}}_{{\rm{v}}}=\frac{1}{2}\mathop{\sum }\limits_{i=1}^{3}\mathop{\sum }\limits_{j=1}^{3}{[(\frac{\partial {{\boldsymbol{v}}}_{{\boldsymbol{i}}}}{\partial {{\boldsymbol{x}}}_{{\boldsymbol{j}}}}+\frac{\partial {{\boldsymbol{v}}}_{{\boldsymbol{j}}}}{\partial {{\boldsymbol{x}}}_{{\boldsymbol{i}}}})-\frac{2}{3}(\nabla .{\boldsymbol{v}}){{\boldsymbol{\delta }}}_{{\boldsymbol{ij}}}]}^{2},$$

2 $$\{\begin{array}{c}{{\boldsymbol{\delta }}}_{{\boldsymbol{ij}}}=1,\,if\,i=j\\ {{\boldsymbol{\delta }}}_{{\boldsymbol{ij}}}=0,\,if\,i\ne j\end{array}\,[{s}^{-2}]$$

Where *ν*_*i*_ (m/s) is the velocity component along the i spatial direction, *x*_*i*_ is the unit vector along i direction, ∇.*ν* is the divergence of the velocity field, and *δ*_*ij*_ is the Kronecker delta. Subsequently, the volumetric rate of VEL at a time instance *EL*_*t*_ (Watt) can be derived by integrating Φ_v_ over the volume domain:

3 $$\dot{E{L}_{t}}=\mu \mathop{\sum }\limits_{i=1}^{number\,of\,voxels}{{\rm{\Phi }}}_{{\rm{v}}}{V}_{i}\,({\rm{Watt}}\,(W))$$

Where *μ* is the dynamic viscosity of fluid (Pa·s) and *V*_*i*_ is the volume element (m^3^). Total VEL (J) in a cardiac cycle was then calculated by integrating ${\mathop{EL}\limits^{\cdot }}_{t}$ over time (s).

#### Vortex detection by Q-criterion

Q-criterion was used to identify vortical structures in the RV to eliminate the contributions of high shear boundary regions^6,14^. In principle, velocity gradient tensor $\nabla {\boldsymbol{v}}$ is decomposed into rate-of-strain tensor ${\rm{S}}$ and vorticity tensor ${\rm{\Omega }}$:

4 $$\nabla {\boldsymbol{v}}={\rm{S}}+{\rm{\Omega }}=\frac{1}{2}[\nabla {\boldsymbol{v}}+{(\nabla {\boldsymbol{v}})}^{{\rm{T}}}]+\frac{1}{2}[\nabla {\boldsymbol{v}}-{(\nabla {\boldsymbol{v}})}^{{\rm{T}}}]$$

*Q* is then defined as:

5 $$Q=\frac{1}{2}[{|{\rm{\Omega }}|}^{2}-{|S|}^{2}]$$

Vortical structure is identified as regions where rotation rate dominates strain rate, i.e., *Q* > 0. In this study, a small positive value of Q = 0.00012 was selected as the threshold value. Vortex and swirling structures were visualized by plotting the iso-surfaces with Q = 0.00012. To quantitatively assess vortical flows, vorticity magnitude can be computed as the magnitude of the 3D vorticity vector:

6 $$|\overrightarrow{\omega }|=\sqrt{{(\frac{\partial {v}_{z}}{\partial y}-\frac{\partial {v}_{y}}{\partial z})}^{2}+{(\frac{\partial {v}_{x}}{\partial z}-\frac{\partial {v}_{z}}{\partial x})}^{2}+{(\frac{\partial {v}_{y}}{\partial x}-\frac{\partial {v}_{x}}{\partial y})}^{2}}\,\,\,\,({s}^{-1})$$

#### 3D principal Reynolds shear stress (PRSS)

Reynolds shear stress (RSS) represents the momentum transfer due to random velocity fluctuations in a turbulent flow regime and has been used as an indicator for blood damage risk^21^. To calculate principal RSS (PRSS), Reynolds decomposition was conducted to determine *u*′, *v*′ and *w*′, which are the instantaneous velocity fluctuation components in *x*, *y* and *z* directions, respectively. The Reynolds shear stress term across (*I*, *j*) plane was calculated as: ${\bar{\tau }}_{ij}^{n}=-\rho \overline{{u^{\prime} }_{i}}{u^{\prime} }_{j}$, with *ρ* being the fluid density, and ${u^{\prime} }_{i}$ the instantaneous velocity fluctuation component in the *i* direction.

Three principal normal stresses ${\bar{\sigma }}_{p1}^{n}$, ${\bar{\sigma }}_{p2}^{n}$ and ${\bar{\sigma }}_{p3}^{n}\,\,$at every point in the fluid domain were derived as eigenvalues of the Reynolds stress tensor:

7 $$|\begin{array}{ccc}{\bar{\sigma }}_{p}^{n}-{\bar{\tau }}_{11}^{n} & -{\bar{\tau }}_{12}^{n} & -{\bar{\tau }}_{13}^{n}\\ -{\bar{\tau }}_{21}^{n} & {\bar{\sigma }}_{p}^{n}-{\bar{\tau }}_{22}^{n} & -{\bar{\tau }}_{23}^{n}\\ -{\bar{\tau }}_{31}^{n} & -{\bar{\tau }}_{32}^{n} & {\bar{\sigma }}_{p}^{n}-{\bar{\tau }}_{33}^{n}\end{array}|=0$$

The three potential maximum shear stresses were then calculated as:

8 $${\bar{\sigma }}_{pij}^{n}=\frac{1}{2}|{\bar{\sigma }}_{pi}^{n}-{\bar{\sigma }}_{pj}^{n}|$$

The largest value among the three computed ${\bar{\sigma }}_{pij}^{n}$ was taken as the largest time-averaged shear stress ${\tau }_{max}^{n}$ at a point in the fluid domain. Thus, ${\tau }_{max}^{n}$ values were compared across the whole fluid domain to determine the largest PRSS at a time instant for that particular region. Since peak E-wave time instant is associated with high inflow velocities and thus likely leads to high levels of PRSS, the above algorithm was used to detect the maximal 3D PRSS inside the RV domain at this particular time instant.

## Results

To quantify the change in overall CO after Kay repair, additional six cases were included in the flow loop experiment. Thus, a total of nine cases were tested in the flow loop, for which the CO was assessed before (FTR state) and after Kay bicuspidization repair. A paired t-test was conducted using R software to determine if the change in CO among the cases is statistically significant. The results of the paired t-test showed that the increase observed in CO following bicuspidization was significant (N = 9, paired t-test, p = 0.00499). The CO data for these cases can be found in Supplementary information (Table S1).

Flow and pressure parameters of the two main valve groups used for area assessment and PIV experiment are depicted in Table 1. Assessment of area metrics in both atrial and ventricular views yielded relatively consistent results. Results in both views were averaged to give the final area parameters of the valves shown in Table 2. Overall, regurgitation was markedly decreased post-repair, with the regurgitant area reduction ranging from 75% to 97% among the valves. Bicuspidization repair substantially reduced TV annulus area and TV opening area for all the valves. However, TV opening area was reduced to a greater extent (up to approximately 57%) as compared to the reduction in annulus area for all three valves tested. As a result, the ratio of TV opening area to TV annulus area dropped for all the valves after bicuspidization.

**Table 2:** Area assessments of the valves.

	Annulus area (cm^2^)	Opening area (cm^2^)	Opening/Annulus area (%)	Regurgitant area (cm^2^)	Annulus area reduction (%)	Opening area reduction (%)	Regurgitant area reduction (%)
Valve 1a	8.51	4.75	55.82	0.80	—	—	—
Valve 1b	7.42	3.37	45.42	0.03	12.81	29.05	96.25
Valve 2a	9.72	6.38	65.64	1.50	—	—	—
Valve 2b	5.94	3.19	53.70	0.37	38.89	50.00	75.33
Valve 3a	11.62	7.58	65.23	1.49	—	—	—
Valve 3b	6.08	3.28	53.95	0.04	47.68	56.73	97.32

### Energy analysis

**Figure 6:**
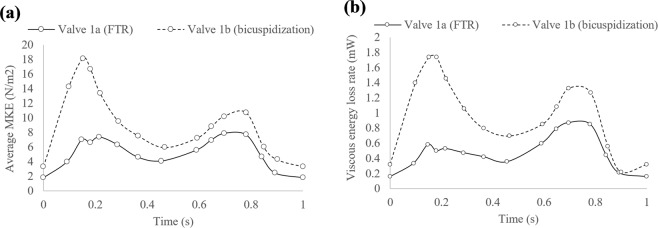
(**a**) Average MKE (N/m^2^), and (**b**) VEL rate (mW) in the RV of Valve 1 over one cycle (starting from early diastole) before and after bicuspidization treatment.

Average MKE inside the RV was plotted over the 14 time-steps in the cardiac cycle for Valve 1 (Fig. 6a). Mean values were calculated for the two valve groups (Table 3). There are generally two instances when average MKE peaked during a cardiac cycle: (i) during diastole and (ii) during systole, as illustrated in Fig. 6. For all valves studied, performing bicuspidization leveraged the total average MKE inside the RV, with the mean percentage increase of 64.15%. The peak diastolic average MKE was prominently increased by 152.72% for the post-repair valve group, whereas the peak systolic average MKE was not consistently increased for all the valves. Interestingly, repair treatment altered the pattern of MKE inside the RV over a cardiac cycle. In valve group (a), the maximal average MKE inside RV was consistently found during systole, whereas for valve group (b), peak diastolic average MKE is the maximal value detected in a cycle. As a result, the ratio of peak diastolic/systolic average MKE increased to 1.38 ± 0.32 after repair treatment as compared to only 0.67 ± 0.27 in the FTR condition.

**Table 3:** Energy and vortex parameters of the two valve groups.

	FTR (n = 3)	Bicuspidization (n = 3)	Percentage increase in mean values (%)
Total average MKE in one cycle (N/m^2^)	62.53 ± 14.08	102.64 ± 33.13	64.15
Peak diastolic average MKE (N/m^2^) (Peak MKE_dias_)	4.97 ± 2.08	12.56 ± 5.42	152.72
Peak systolic average MKE (N/m^2^) (Peak MKE_sys_)	7.62 ± 1.92	8.83 ± 1.86	15.93
Peak MKE_dias_/Peak MKE_sys_ ratio	0.67 ± 0.27	1.38 ± 0.32	—
Total VEL in one cycle (mJ)	0.42 ± 0.08	0.69 ± 0.24	64.29
Peak diastolic VEL rate (mW)	0.35 ± 0.01	1.15 ± 0.57	228.57
Total of mean RV vorticity magnitude in a cycle (s^−1^)	146.14 ± 20.10	205.54 ± 36.65	40.65
Total of mean Q-thresholded vorticity magnitude in a cycle (s^−1^)	358.34 ± 48.87	432.43 ± 67.76	20.68

Results are represented as mean ± SD.

FTR = functional tricuspid regurgitation; MKE = mechanical kinetic energy; RV = right ventricle; VEL = viscous energy loss.

Regarding viscous energy loss, the general trend and shape of the VEL rate curves typically resemble the MKE plots. One example of VEL rate inside RV over a cardiac cycle (Valve 1) is shown in Fig. 6b. Bicuspidization repair elevated the RV viscous loss levels in all cases. Mean increment in total VEL in one cycle after bicuspidization repair is 64.29% among the valves (Table 3). In particular, peak diastolic VEL rate was consistently and substantially increased post-repair for all valves tested, with the mean increase of 228.57%.

### Flow and vortex description

**Figure 7:**
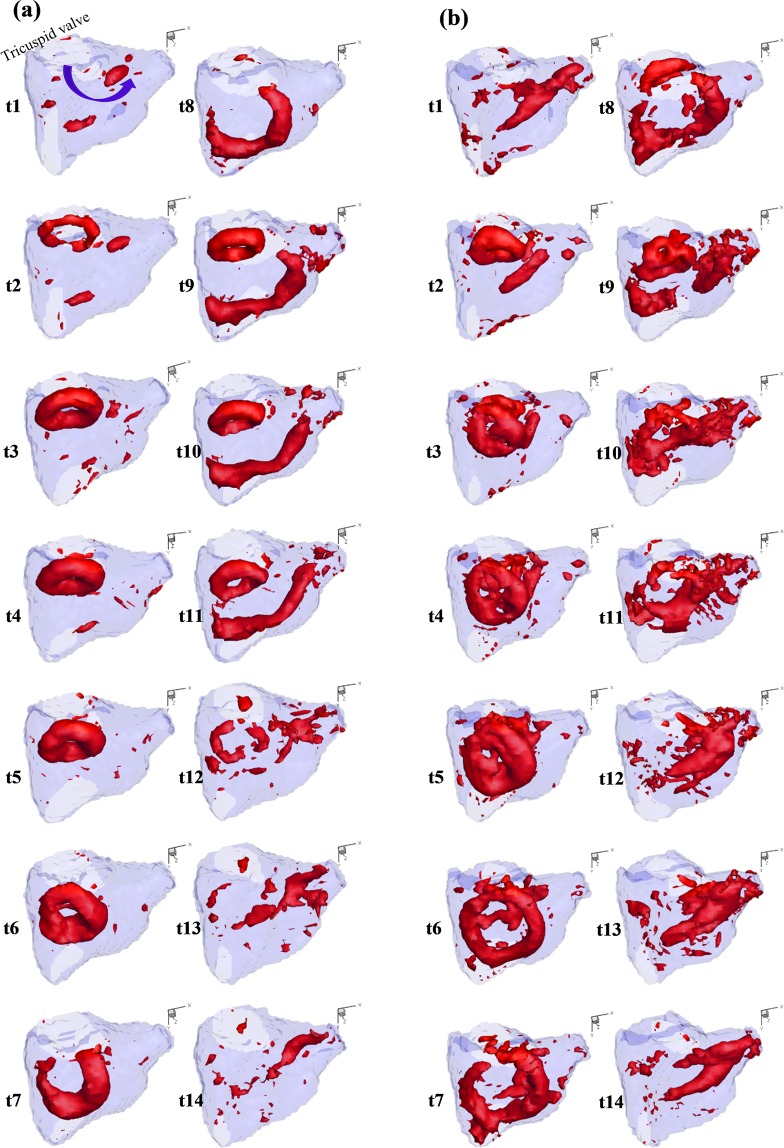
Vortex formation and evolution over one cycle for Valve 1 before (**a**) and after bicuspidization treatment (**b**).

Figure 7 depicts the formation and evolution of vortex inside the RV for Valve 1a (FTR) and Valve 1b (bicuspidization repair).

### Valve 1a (severe FTR)

**Figure 8:**
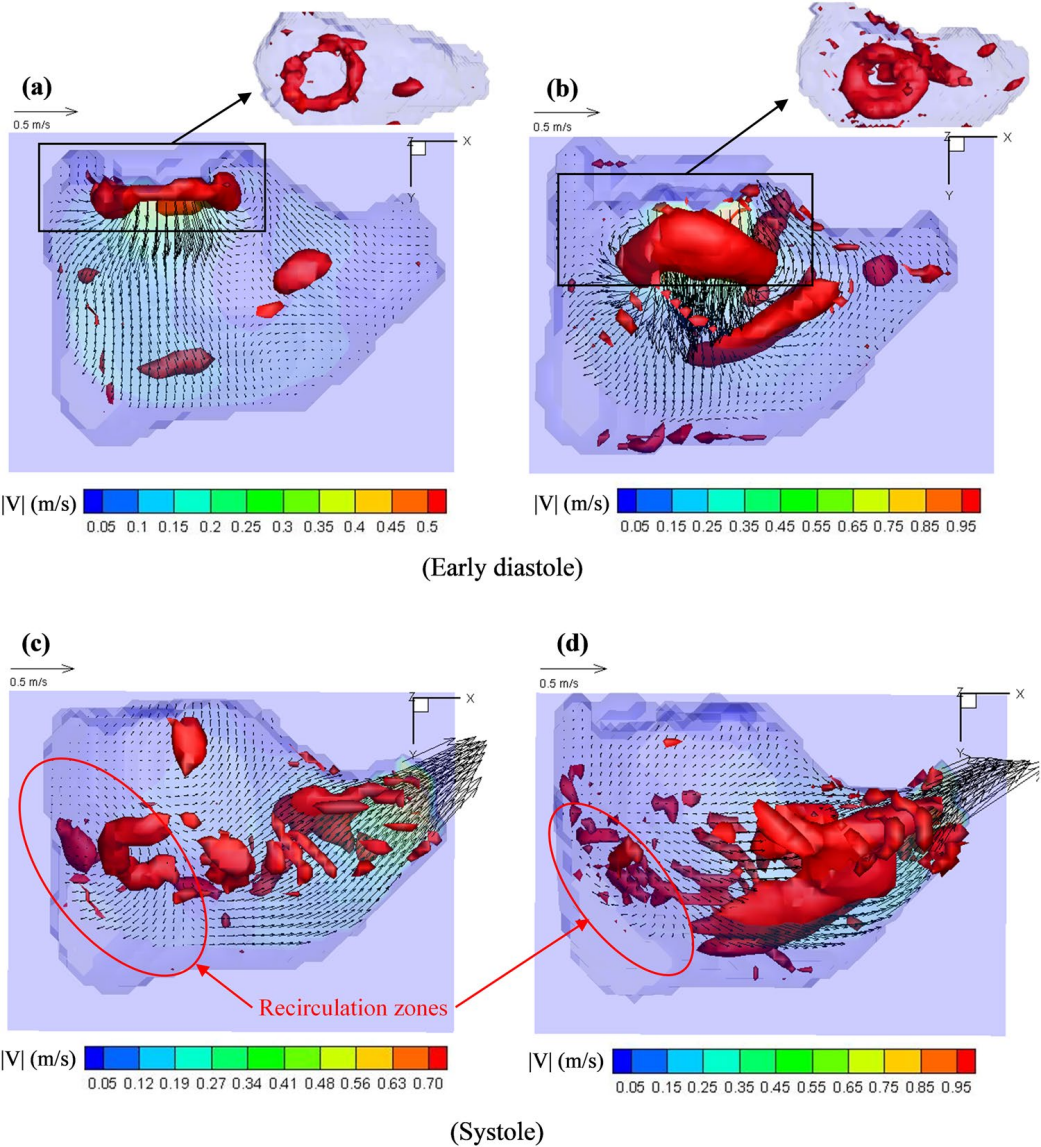
Velocity vector fields of middle plane cutting through the tricuspid orifice and pulmonary outlet of Valve 1a ((**a**,**c**)) and Valve 1b ((**b**,**d**)) at early diastole and during systole.

As presented in Fig. 7a, a thin vortex ring formed past the tricuspid orifice in early diastole. The atrial view at time-point 2 (Fig. 8) shows the circular and symmetrical shape of the vortex ring. The structure continued to evolve and became thicker as it propagated into the RV cavity. At time-point 6, the ring resided roughly in the middle of the RV cavity with the whole ring structure still intact. At time-point 7, the septum part of the ring was maintained while the anterior part was dissipated possibly due to the interaction with the anterior wall. Subsequently, a similar vortex ring formed during late diastole, while the remnants of previous vortical structure were elongated towards the pulmonary outlet. By late systole, no coherent structure could be observed and there were only vorticity residuals inside the RV cavity, especially near the pulmonary outlet.

### Valve 1b (after bicuspidization repair)

As seen in Fig. 7b, early diastolic inflow created a vortex ring formed past the bicuspid orifice. Compared to similar structure observed for Valve 1a at the same time-point, the ring was constrained in a smaller circumferential space albeit with thicker configuration, thus occupying larger iso-surface volumes. Moreover, the vortex assumed a slightly 3D saddle shape. Subsequently, the vortex ring increased in size and moved further into the RV cavity. Unlike in FTR situation, the vortex structure in this case took up much larger RV space as it evolved. At the same time, the vortex core was more irregular and could no longer be identified as a single compact vortex ring. In addition, the direction of propagation is more biased towards the lateral-anterior side of RV compared to previous case. By the time the vortical structure was in the middle of the RV, it had taken up most of the intraventricular space.

During late diastole, a more planar but incomplete (discontinuous at the anterior-septal side) vortex ring formed past the orifice. Similar to FTR case, the vortical structure formed during early diastole dissipated considerably at the lateral-anterior side. However, part of the septal vortical structure was also dissipated due to the impingement onto the septal wall. As a result, this vortical structure broke into halves (time-points 8 & 9). As the newly formed vortex ring proceeded into RV cavity, the part of previous vortical structures near the posterial wall continued to dissipate, while the other half extended towards the pulmonary outlet. During systole when vortical ring structure could no longer be observed, there was a large volume of vorticity contour near the outlet, in the form of elongated structures that continued to exist into early diastole. Compared to Valve 1a, vorticity contours of Valve 1b showed a more complex evolution behaviour with lots of vorticity residuals which did not belong to any compact vortical structure.

### Velocity vector fields

Figure 8 shows the velocity field inside the RV (for Valve 1 before and after repair) at the middle plane cutting through the tricuspid orifice and pulmonary outlet at early diastole and peak systole. For Valve 1a (FTR), early inflow was distributed quite homogenously along the tricuspid inlet accompanied by the thin, compact, and symmetrical vortex ring. Maximal inflow velocity recorded was 0.61 m/s for Valve 1a, 0.45 m/s for Valve 2a, and 0.43 m/s for Valve 3a. After bicuspidization, the inflow was constrained into a stronger and narrower central jet, forming a thicker and nonplanar vortex ring. Maximal inflow velocity increased to 1.00 m/s for Valve 1b, 0.93 m/s for Valve 2b, and 0.86 m/s for Valve 3b.

At peak systole, scattered flow patterns were found in some RV regions for both valve groups where velocity vectors were not directed towards the outlet (Fig. 8c,d). Specifically, certain regions in the vicinity of the TV remained with the velocity vectors directed back toward the tricuspid orifice even after repair, indicating residual TR. In addition, part of the RV blood near the posterior wall was not effectively directed towards the pulmonary outlet in both cases.

### Total vorticity magnitude

The mean vorticity magnitude over the whole RV domain as well as Q-thresholded vortical structures were quantified for each time-point and summed over the cardiac cycle. These values were averaged for both groups and are presented in Table 3. Bicuspidization repair subjected the RV domain to increased swirling motions in general, characterized by the percentage increases in both total of mean RV vorticity magnitude (40.65% increase) and total of mean Q-thresholded vorticity magnitude (20.68% increase) in a cycle.

### Turbulent shear

It is expected that the reduced opening area and elevated inflow velocities increase the level of turbulent shear stress. This is supported by our results which showed that bicuspidization repair elevated the maximal PRSS detected inside the RV domain at the peak E-wave time instant for all three cases studied. The maximal PRSS observed at peak E-wave for the FTR valves was 21.66 N/m^2^ for Valve 1a, 27.26 N/m^2^ for Valve 2a, and 27.23 N/m^2^ for Valve 3a, whereas these values increased to 32.55 N/m^2^ for Valve 1b, 53.04 N/m^2^ for Valve 2b, and 46.14 N/m^2^ for Valve 3b. Nevertheless, all the values are well below the threshold stated in the literature for blood hemolysis risk^21^ (800 N/m^2^).

It is also known that the platelet activation could be induced by the combination of high shear stress and large exposure times^22,23^. A platelet activation parameter was previously formulated as the integral of shear stress and time^24^. This concept was subsequently applied to assess the platelet activation tendency of fluid going through high shear regions by estimating the shear stress-time product^25^. In the present study, the time for a particle moving through the tricuspid orifice with high E-wave inflow velocities was estimated to be <0.0230 s for Valve 1b, <0.0133 s for Valve 2b, and 0.0243 s for Valve 3b. This corresponded to the shear stress-time product of <0.7487 Pa.s for Valve 1b, <0.7041 Pa.s for Valve 2b, and 1.1216 Pa.s for Valve 3b. These values are below 3.5 Pa.s which has been suggested for procoagulant platelet factor 3 release^23^.

## Discussion

Although bicuspidization was able to correct for TR, the repair also reduced TV annulus area and especially TV opening area substantially. Moreover, bicuspidization alone does not necessarily result in complete eradication of regurgitation (Table 2). The extent of regurgitant area reduction differed among three samples, which was likely attributed to the variation in individual TV morphology and dimension. The same variation might be expected in clinical scenarios, although in practice clinicians can have the choice to influence this extent of TR by modifying the technique to allow cinching of more annulus portion. Nevertheless, the plication of the posterior annulus might exert additional stretching on the remaining anterior and septal leaflets, altering their morphology from their original states. Although these two leaflets were brought closer and thus systolic coaptation was enhanced, their diastolic opening motions were also more constrained. This might explain the greater percentage reduction in TV opening area compared to the reduction in TV annulus area. As a result, it is likely that the gain in CO by regurgitation elimination was offset partly by the limited inflow caused by the reduced TV opening area. The current results showed that although bicuspidization consistently improved CO among all cases tested, the increment was generally small (Table 1 & Table S1, Supplementary information). Further studies with more samples and possible anatomical variation of various elements involved in the pathology are needed to better understand the effect of bicuspidization on CO. In a previous study, Vismara *et al*.^26^ performed different procedural variations of edge-to-edge repair on an *ex vivo* porcine model of FTR; the outcomes in terms of CO and pressure were found to be strongly dependent on the pair of leaflets grasped as well as the position of clips. Indeed, many of the variations resulted in marginal if not detrimental effects on the CO recovery. Thus, while certain repair techniques can diminish regurgitation and resolve the elevated atrial and venous pressures, their impact on CO recovery might be deemed unsatisfactory.

Prior studies have demonstrated the altered ventricular KE levels and patterns in pathological conditions compared to healthy controls^27–30^. In the current study, bicuspidization repair enhanced the KE levels of RV in general. Especially, the repair altered the pattern of MKE inside the RV over a cardiac cycle in comparison with that observed in FTR state. While FTR valves exhibited highest KE during systole, similar to that reported for healthy RV^31^, maximal KE for the repaired valves was shifted to early diastole. This implied that the physiological flow pattern was not restored with repair. The prominently increased diastolic MKE observed post-repair is likely attributed to the abnormal high velocity inflow pattern which is not the case in healthy RV or in FTR pathology. As the valve opening area is considerably reduced, blood is subjected to higher velocity over a smaller inflow area – resembling forward stenosis to some extent. The peak velocity of FTR valves in diastole ranged from 0.43 m/s to 0.61 m/s. After bicuspidization repair, this value was markedly increased up to 1.00 m/s. In practice, it is not uncommon to record peak trans-tricuspid velocity exceeding 1 m/s in tricuspid stenosis^32^. Theoretically, KE is defined as the work needed to accelerate a given mass of blood from rest to its velocity^33^. Given that the CO did not differ much after repair, it is speculated that higher KE levels post-repair might indicate that greater work was necessary to transfer the fluid from inlet to outlet. Indeed, the large increase in VEL levels post-repair also implied that higher degree of the work done to accelerate the fluid was lost to heat inside the RV after repair, as compared to the energy loss observed before repair. Nevertheless, it is also noted that in this scenario, the RV model exhibited severe dilatation with reduced contraction, thus an optimal flow pattern with lower energy expenditure yet higher output might not be possible with any other treatment approaches. Increasing number of studies have proposed MKE as a potential cardiac marker and driver in disease progression or morphologic changes. Altered intraventricular energy levels have been suggested to influence the pressure gradients and the subsequent myocardial remodelling^7,27^. Moreover, any subtle inefficiency in energy expenditures might contribute to cardiac failure over extended time^28^. In this perspective, it is important to quantify MKE under different pathologies and procedures. Further studies relating the MKE changes and clinical consequences are necessary and could be utilized to guide future medical and surgical interventions.

The overall vortex phenomenon was mostly preserved after repair. In general, two vortex rings formed past the valve orifice during early and late diastole and evolved as they propagated into the RV cavity. The perseverance of vortical structures in the RV space as they moved past the tricuspid orifice could be the result of the RV dilatation, leading to more cylindrical expanded configuration. Subsequently, the vortical structures dissipated partly after interaction with RV wall and elongated towards the outlet during systole. This possibly represented the re-organization of vortical structures into the helical patterns as the blood exited through a gradually narrowing pathway. There are, however, some distinctive characteristics between the two valve groups. Post-procedural vorticity contours typically exhibited more isolated vorticity residuals. In addition, vortical cores inside the RV post-repair acquired more irregular structures as compared to the compact and relatively symmetrical nature of those formed inside the RV before repair. Given that the tricuspid orifice was modified to be smaller and more elliptical, along with the transformation of leaflet morphology to be mitral-like configuration, altered inflow dynamics and vortex formation were expected. Quantitatively, bicuspidization subjected RV domain to augmented levels of swirling motions as indicated by vorticity magnitude calculations. Post-procedural vortical structures also took up a larger RV domain as compared to FTR cases. On the one hand, strong circulation in larger RV domain might reduce the risk of mural-thrombus formation^34–37^. Moreover, when the dilated RV typically loses its normal contractibility in pathological situations such as severe FTR, the possible contribution of more dominant vortex and helical structures to fluid transfer could become particularly relevant. On the other hand, stronger vortical motion accompanied by increased irregular structures and vorticity residuals could imply more chaotic interactions and loss of energy due to viscous dissipation and turbulence. Various studies have demonstrated the correlation between disrupted natural vortex patterns and elevated viscous energy loss^20,38^. Likewise, the current results showed substantial increase in VEL inside the RV after repair, especially during diastolic filling, implying the increased viscous loss associated with more irregular vortex structures. In addition, the modified vortex pattern might subject the RV to altered shear stress levels with increased regions exposed to elevated shear, potentially leading to long-term adaptation consequences. In terms of turbulent shear, the repair increased the maximal PRSS observed inside the RV at peak E-wave for all three valves tested. Nevertheless, these values were well below the hemolytic RSS threshold^21^. Likewise, estimated values of shear-time integral for the repaired valves were below the threshold indicated for platelet activation^23^. Thus, it is unlikely that blood components would be subjected to noticeable risk of damage due to turbulent shear even after repair.

In the LV, preservation of vortex structure have been reported with certain MV repair strategies^39,40^, while significant alteration to complete elimination of vortical structures were observed in other approaches^38–42^. In this study, although bicuspidization repair increased viscous loss and led to more irregular vorticity residuals, the main vortex structures were generally maintained. Nevertheless, both FTR and post-repair RV flow domains exhibited non-optimal flow pattern, including the varying extent of regurgitation and recirculation regions during systole. While the use of annuloplasty in conjunction with bicuspidization repair might help in eliminating regurgitation, the influence of further reduced orifice area on hemodynamic parameters and valvular area metrics is unknown and deserves further investigation. Given the reduced RV contractibility along with the dilated RV morphology, attaining the ideal fluid transfer behaviour might be difficult with any strategy.

Currently, transcathether technology is emerging as an attractive treatment option for high-risk patients. Nevertheless, TV interventional therapies are still at its early stages of development compared to other valvular treatments. Among different percutaneous approaches, Mitralign system (Mitralign, Tewksbury, MA, USA) can reproduce Kay bicuspidization surgical repair. In summary, the device is used to place pledget sutures by means of a trans-jugular approach^43^. Insulated radiofrequency wires are typically positioned 2 to 5 mm from the base of the posterior leaflet and within the annulus, at the postero-anterior commissure and septo-posterior commissure. Once these wires are through the annulus, pledgets can be advanced over the wires and cinched to the corresponding anatomical sites on the annulus portion. The sutures connecting the two pair of pledgets are then drawn together and locked to plicate the posterior annulus. This approach was applied in first-in-man procedure in 2014 and an early feasibility study is currently ongoing (the SCOUT study – Early Feasibility of the Mitralign Tricuspid Valve Annuloplasty System)^4^. Although feasibility and successful implantation have been shown possible in a number of patients, durable results post-treatment are currently uncertain. Moreover, a ‘partial’ correction of TR could be considered to avoid the hemodynamic burden of acute complete correction of TR to the RV^4^, although long-term efficacy of this approach remains unclear. Results from the current study imply that complete bicuspidization might lead to some extent of forward stenosis due to the restricted leaflet opening area, potentially limiting the cardiac output recovery. How a partial correction approach could influence the cardiac output and other hemodynamic parameters remains unknown and deserves further research.

## Limitations

The experimental system was subjected to several intrinsic constraints of common mock simulators, including the lack of torsion and strong ventricular wall dynamic motion and the absence of LV-RV interaction. Nevertheless, it has been shown that twisting and rotational motions do not contribute significantly to RV contraction in contrast to the LV^44^. Moreover, in the setting of severe RV enlargement, the intraventricular septum is shifted leftward^44,45^ leading to a more spherical RV configuration. It is also likely that the dynamic motion of the RV would be reduced in comparison to a healthy heart, due to the combination of long-standing RV dilatation and aging factor which is common in severe TR population. Thus, these factors would partly justify the characteristics of the present *ex vivo* system.

In the present study, the trans-tricuspid flow waveform post-repair was not reported as an indicator for the efficacy of resolving TR. During TV closure, the total amount of back flow was contributed by both the closing volume due to the leaflets closing motion and the pathological regurgitant volume caused by the incomplete coaptation of the leaflets^46,47^. It has been established in the literature that detection of true pathological regurgitant volume of the TV is not straightforward due to the contribution of closing volume and the fact that the majority of the population has some degree of “physiologic” TR^46^. In the current system, the flow sensor was clamped onto the plastic tubing upstream of the RV chamber where the TV was housed. Since there was an unavoidable distance between the sensor and the exact location of the valve, the signals received by the sensor seem to mostly reflect the closing volume and might not accurately reflect the regurgitant volume. Hence, the regurgitant flow is not detected without being overshadowed by the closing volume. Other flow measurement methods such as those that can assess directly the regurgitant jet inside the fluid^15,46,48^ might be more useful in this scenario to assess the true TR volume or severity. Hence, trans-tricuspid flow curve was mainly used as a mean for validation of systemic hemodynamic condition rather than a tool to quantify accurate TR volume in the present study.

The limited number of samples in this study could impose constraint in the validity of results in more clinical situations. Nevertheless, the present study aims to address the efficacy of bicuspidization in a severe FTR scenario which includes all elements of RV dilatation, PM displacements and TV annular dilatation. While the existence and degree of geometrical modification for each of these components might vary clinically, it is expected that severe and late stage of FTR would exhibit all the characteristics addressed in this study^49^. Future studies involving more samples and geometrical variations will be conducted to warrant the validity of current findings.

In addition, only Kay bicuspidization approach was investigated in this study. Several other treatments are also common in clinical practice which require further investigations, including annuloplasty, edge-to-edge repair, as well as replacement with prosthetic valves. Furthermore, since the *ex vivo* system did not emulate outflow obstruction, this study only considers cases of severe FTR without pulmonary hypertension.

## Conclusions

In this study, TV area and RV hemodynamic characteristics before and after Kay bicuspidization repair in a severe FTR situation exhibiting both severe RV and TA dilatations were investigated for the first time in a novel *ex vivo* right heart simulator by means of stereo-scopic PIV. Results indicated that the repair led to significant increase in cardiac output although the overall increment due to this approach alone was generally small, possibly due to existence of residual TR and the large reduction in TV opening area. The repair elevated kinetic energy of RV fluid especially during diastolic filling. Main flow and vortex structures were generally maintained post-procedural. However, more irregular vortical structures and more isolated vorticity residuals were observed inside the RV after repair. The enhanced swirling motion in a larger RV domain after repair might reduce the risk of mural-thrombus formation and potentially aid in fluid redirection. Nevertheless, the relatively more complex vortex formation led to augmented friction-driven interactions indicated by the observed increase in viscous energy loss, especially during diastolic filling. Further investigations are needed to assess the efficacy of tricuspid repairs in more stages of FTR as well as to link the difference in vortex patterns and energy metrics with potential clinical outcomes. This study provides better understanding of how RV hemodynamic conditions are altered with TV repair which could allow better treatment design and planning in the future.

## Supplementary information

S1

## Data Availability

The datasets generated during and/or analysed during the current study are included in this article (and its Supplementary Information file), and are available from the corresponding author(s) on reasonable request.
