# Supplementary material for: Ex vivo assessment of bicuspidization repair in treating severe functional tricuspid regurgitation: a stereo-scopic PIV study
Source: Sci Rep. 2019 Aug 8;9:11504. doi: 10.1038/s41598-019-47873-3 (PMC6687742; doi:10.1038/s41598-019-47873-3)
Supplement: Supplementary file 1 — S1 [file 41598_2019_47873_MOESM1_ESM.pdf]

Ex vivo assessment of bicuspidization repair in treating severe functional tricuspid regurgitation: a stereo-scopic PIV study

Yen Ngoc Nguyen[1], Foad Kabinejadian[2], Munirah Ismail[3], William Kok-Fai Kong[4], Edgar Lik Wui Tay[4 *], Hwa Liang Leo[1,5 *]

[1] Department of Biomedical Engineering, National University of Singapore, Singapore

[2] Department of Biomedical Engineering, Tulane University, New Orleans, LA 70112, USA

[3] School of Mechanical and Aerospace Engineering, Nanyang Technological University, Singapore

[4] Department of Cardiology, National University Heart Centre, Singapore

[5] NUS Graduate School for Integrative Sciences and Engineering, National University of Singapore, Singapore

[4 *] Address correspondence to this author at Department of Cardiology, National University Heart Centre, Singapore; Email: Edgar_Tay@nus.edu.sg

[1,5 *] Address correspondence to this author at Department of Biomedical Engineering, National University of Singapore, Singapore; Tel: +65-6516-5608; Email: bielhl@nus.edu.sg

SUPPLEMENTARY INFORMATION

TABLE S1 Cardiac output of the valves

Valve No.	**1**	**2**	**3**	**4**	**5**	**6**	**7**	**8**	**9**	**Average**	**SD**
FTR (L/min)	3.9	3.9	3.9	3.7	4	3.8	3.7	3.9	3.8	3.84	0.10
Bicuspid (L/min)	4.2	4.1	4.1	4	4	3.9	3.8	4	3.8	3.99	0.14
Change (L/min)	0.3	0.2	0.2	0.3	0	0.1	0.1	0.1	0	0.14	0.11

FTR = functional tricuspid regurgitation; SD = standard deviation.
